# Diamond/Porous Titanium Nitride Electrodes With Superior Electrochemical Performance for Neural Interfacing

**DOI:** 10.3389/fbioe.2018.00171

**Published:** 2018-11-15

**Authors:** Suzan Meijs, Matthew McDonald, Søren Sørensen, Kristian Rechendorff, Ladislav Fekete, Ladislav Klimša, Václav Petrák, Nico Rijkhoff, Andrew Taylor, Miloš Nesládek, Cristian P. Pennisi

**Affiliations:** ^1^SMI, Department of Health, Science and Technology, Aalborg University, Aalborg, Denmark; ^2^Institute for Materials Research, University of Hasselt, Diepenbeek, Belgium; ^3^Materials Division, Danish Technological Institute, Århus, Denmark; ^4^Department of Functional Materials, Institute of Physics of the Czech Academy of Sciences, Prague, Czechia; ^5^Laboratory for Stem Cell Research, Department of Health Science and Technology, Aalborg University, Aalborg, Denmark

**Keywords:** neural prosthesis, neural interfaces, implantable electrodes, electrical stimulation, boron-doped diamond, porous diamond, titanium nitride, electrochemistry

## Abstract

Robust devices for chronic neural stimulation demand electrode materials which exhibit high charge injection (*Q*_inj_) capacity and long-term stability. Boron-doped diamond (BDD) electrodes have shown promise for neural stimulation applications, but their practical applications remain limited due to the poor charge transfer capability of diamond. In this work, we present an attractive approach to produce BDD electrodes with exceptionally high surface area using porous titanium nitride (TiN) as interlayer template. The TiN deposition parameters were systematically varied to fabricate a range of porous electrodes, which were subsequently coated by a BDD thin-film. The electrodes were investigated by surface analysis methods and electrochemical techniques before and after BDD deposition. Cyclic voltammetry (CV) measurements showed a wide potential window in saline solution (between −1.3 and 1.2 V vs. Ag/AgCl). Electrodes with the highest thickness and porosity exhibited the lowest impedance magnitude and a charge storage capacity (CSC) of 253 mC/cm^2^, which largely exceeds the values previously reported for porous BDD electrodes. Electrodes with relatively thinner and less porous coatings displayed the highest pulsing capacitances (*C*_pulse_), which would be more favorable for stimulation applications. Although BDD/TiN electrodes displayed a higher impedance magnitude and a lower *C*_pulse_ as compared to the bare TiN electrodes, the wider potential window likely allows for higher *Q*_inj_ without reaching unsafe potentials. The remarkable reduction in the impedance and improvement in the charge transfer capacity, together with the known properties of BDD films, makes this type of coating as an ideal candidate for development of reliable devices for chronic neural interfacing.

## Introduction

Nanocrystalline diamond films synthesized by means of chemical vapor deposition (CVD) represent a unique class of materials with outstanding physical and chemical properties, including superior hardness and the ability to resist extreme corrosive environments (Williams, 2011). Besides these features, electrically conductive boron-doped diamond (BDD) exhibits a wide potential window and low background currents, which make it a fascinating material for electrochemical applications (Rao and Fujishima, 2000). During the last few decades, BDD has been employed for the fabrication of electrodes for a wide range of applications, including electroanalysis (Compton et al., 2003; Suzuki et al., 2007; Schwarzová-Pecková et al., 2017), electrosynthesis (Kraft, 2007; Ivandini and Einaga, 2017; Ashcheulov et al., 2018), and biosensing (Vermeeren et al., 2009; Zhou and Zhi, 2009; Qureshi et al., 2010; Svítková et al., 2016). More recently, BDD attracted attention as electrode material for neurochemical sensing, neural recording, and neural stimulation applications, both *in vitro* and *in vivo* (Hébert et al., 2014b; Garrett et al., 2016). Studies have shown that BDD microelectrodes are suitable for the measurement of bioelectric potentials from cultured mammalian neural cells (Ariano et al., 2005; McDonald et al., 2017) and from neural tissue in acute settings (Ho-Yin et al., 2009). BDD holds also great promise for the fabrication of implantable electrodes for chronic application, as the material exhibits extraordinary physical stability, biocompatibility, and resistance to protein biofouling *in vivo* (Alcaide et al., 2016b; Meijs et al., 2016a). However, in contrast to conventional electrode materials, planar BDD films display relatively lower double layer capacitance, and high impedance (Swain, 1994; Alehashem et al., 1995). This is a drawback for neural stimulation applications, as the amount of charge that can be effectively injected through electrodes with relatively small contact sites is quite limited.

Several approaches have been proposed to increase the effective electrochemical area of BDD films as means to boost the amount of charge that could be transferred through the interface. In classical top-down strategies, diamond films are typically etched under a reactive plasma atmosphere to increase their porosity (Yu et al., 2014). In this direction, Kiran et al. have demonstrated successful *in vitro* recording and stimulation of neural preparations using microelectrode arrays (MEAs) comprising “nanograss” BDD contact sites (Kiran et al., 2013). Although this approach has shown to achieve a moderate increase in the electrode capacitance, the fabrication method remains complex and time-consuming, compromising its industrial viability. Alternatively, in bottom-up strategies, a highly porous substrate is used as a template onto which thin diamond films are deposited. Some examples within the various types of porous templates include vertically aligned carbon nanotubes (Hébert et al., 2014a; Zanin et al., 2014), TiO_2_ nanostructures (Siuzdak et al., 2015), and SiO_2_ fibers (Petrák et al., 2017; Vlčková Živcová et al., 2018). Accordingly, BDD electrodes using 3 μm-long vertically aligned carbon nanotubes as an interlayer template have displayed a significant increase in charge storage capacity (CSC) and reduction in the impedance. This improvement in the electrochemical properties allowed successful stimulation and recording of electrical activity in excised mouse hindbrain preparations (Piret et al., 2015). However, integration of carbon nanotubes in implantable neural probes still faces some concerns, due to the risks of long-term cytotoxic effects and the mechanical damage that might occur during implantation (Musa et al., 2012; Liu et al., 2013).

Titanium nitride (TiN) is an attractive material, which can be applied for the fabrication of porous templates with high electrochemical surface area (ESA) by simple physical vapor deposition techniques. Porous TiN coatings have long been employed for pacemaker electrodes and have also been used for fabrication of neural stimulation and recording electrodes (Norlin et al., 2005; Specht et al., 2006; Meijs et al., 2015a). The porosity of TiN films can be easily controlled by adjusting the deposition parameters, such as gas composition, flow rate, and deposition time (Norlin et al., 2005; Cunha et al., 2009). The pores extend deep into the coating, resulting in a high ESA and a high CSC (Cunha et al., 2009). In a preliminary study, we have confirmed the feasibility of fabricating electrodes based on a thin-film BDD deposited on TiN and shown that these electrodes exhibited a relatively high CSC due to the wide potential window typical for BDD (Meijs et al., 2015b).

In this work, the aim is to identify deposition conditions that would allow fabricating BDD electrodes suitable for neural stimulation applications. A range of porous TiN electrodes was fabricated and subsequently deposited with a BDD thin-film. The morphology, quality, and surface properties of the resulting BDD/TiN films were characterized. In addition, we assessed the influence of the underlying TiN film parameters on the electrochemical performance of the electrodes by means of cyclic voltammetry (CV), voltage transient (VT) measurements, and electrochemical impedance spectroscopy (EIS).

## Materials and methods

### Electrode fabrication

The test samples were fabricated using a monopolar Ti_6_Al_4_V electrode pin, which belongs to a system intended for genital nerve stimulation (Martens et al., 2011). Seven types of TiN coatings were evaluated, which were deposited on the electrodes' contact sites by reactive DC magnetron sputtering. Deposition was carried out using an industrial coating unit (CC800, CemeCon AG, Germany) from two Ti targets (88 × 200 mm) with 99.5% purity in a mixed Ar/N_2_ atmosphere. In one set of samples (designated as samples I to V), the N_2_ flow was varied from 30 to 300 standard cubic centimeters per min (sccm), while the deposition time was kept constant at 27.5 × 10^3^ s. In another set of samples (designated as samples III, VI, and VII), the flow rate of N_2_ was kept at 180 sccm while the deposition time was modified. In both cases, the Ar flow was kept constant at 180 sccm.

BDD thin films were synthesized on the TiN layers using an Astex AX6500 microwave plasma enhanced CVD system. The TiN-coated electrodes were first immersed in a 0.33 g/L solution of diamond nanoparticles (3.8 ± 0.7 nm) from Shinshu University to seed the surface for diamond growth. Hydrogen gas with an addition of 1% CH_4_ was added to the chamber at a total flow rate of 500 sccm. Tri-methyl boron was added to the gas as the dopant source, at boron to carbon concentrations of 10,000 ppm. The substrate temperature was maintained at ~750°C by using a pressure of 25 Torr (3.33 kPa) and a microwave power of 2,500 W.

### Surface characterization

The TiN thin-films were investigated using scanning electron microscopy (SEM) (Nova 600, FEI, The Netherlands). Detailed images of all electrodes were recorded at 80,000× magnification. For the assessment of film thickness, flat substrates (10 × 10 mm) obtained from silicon wafers were coated during deposition of each batch of electrodes. The silicon substrates were placed in a manner that ensured an even coating thickness. The coated substrates were subsequently broken and analyzed by cross sectional SEM. The thickness was measured via analysis of the SEM micrographs using Image J (NIH, Bethesda, MD). Each sample was measured at several locations along the cleavage to assess for thickness variations. Only small variations were observed and a unique thickness could unambiguously be assigned to each sample. The surface morphology of the BDD/TiN films was analyzed by a FERA3 GM SEM (Tescan, Czech Republic) with Schottky field emission cathode (FEG-SEM). Images were taken in the high-resolution mode at the accelerating voltage of 5 kV to minimize the interaction volume.

Raman spectroscopy of BDD/TiN films was carried out at room temperature using an InVia Raman Microscope (Renishaw ApS, Denmark) with the following conditions: wavelength = 325 nm, ×40 Olympus objective, 65 μm slits, spot focus, grating = 2,400 L/mm. A high pressure, high temperature Ib single crystal diamond was used as a reference for the sp^3^ Raman peak position.

Topography and surface roughness over a large area (220 × 280 μm^2^) was investigated by an optical profilometer (NewView 7200, ZYGO, Middlefield, CT). In addition, surface roughness and topography over a small area (5 × 5 μm^2^) were investigated by atomic force microscopy (AFM) using a Dimension Icon ambient AFM (Bruker, Germany) in peak force tapping mode using Tap150AL-g tips (BudgetSensors, Innovative Solutions Bulgaria).

### Electrochemical measurements

All electrochemical measurements were carried out in a three-electrode set-up, using the either the TiN or the BDD/TiN electrodes as working electrodes (0.06 cm^2^), a platinum foil counter electrode (50 cm^2^), and a Ag|AgCl reference electrode (1.6 cm^2^). Measurements were performed in Ringer's solution at room temperature.

Cyclic voltammetry was performed by cycling the electrode potential between the water window limits. These limits were determined by increasing and decreasing the electrode potential until an exponentially increasing current was observed using a sweep rate of 0.05 V/s. Measurements were made at 0.05, 0.1, 0.5, and 1.0 V/s; 10 cycles were recorded at each sweep rate. The cathodic CSC of the electrodes was found by calculating the surface area under the zero current axis. The electrochemical surface area to geometrical surface area (ESA/GSA) ratio was calculated by dividing the CSC of the porous coatings by the CSC of the corresponding smooth coating at a sweep rate of 0.05 V/s.

Voltage transient measurements were made using a cathodic-first bipolar symmetric current pulse with an interphase, during which no current was applied. Each phase had a phase width of 200 μs and the duration of the inter-phase was 40 μs. For analysis of the VTs, the OCP was set to 0 V and the IR drop was subtracted. The IR-drop was calculated for each phase by subtracting the potential at 20 μs after pulse cessation from the last data point of the respective phase. The pulsing capacitance (*C*_pulse_) was calculated for each pulse using the following equation:

$$\begin{array}{l} I_{stim}=C_{pulse}\times \frac{dV}{dt} \end{array}$$

where *I*_stim_ is the stimulation current and *dV*/*dt* is the slope of the last 90% of the cathodic phase of the VT. The *C*_pulse_ of the type I TiN electrodes was determined at a current at which safe potential limits were reached. The *C*_pulse_ of the other electrodes was determined at a stimulation current of 20 mA.

Cyclic voltammetry and VT measurements were performed with VersaSTAT 3 potentio-galvanostat (Princeton Applied Research, USA). The impedance spectrum was measured from 0.1 Hz to 100 kHz, five points/decade using a sinusoidal measurement current of 5.0 μA. Impedance spectroscopy was performed using Solartron, Model 1294 in conjunction with 1260 Impedance/gain-phase Analyzer (Solartron Analytical, UK). Linear regression analyses of the CSC and *C*_pulse_-values were performed in Prism 7 (GraphPad Software Inc, La Jolla, CA).

## Results

### Effect of deposition conditions on the surface properties of TiN films

**Figure 1:**
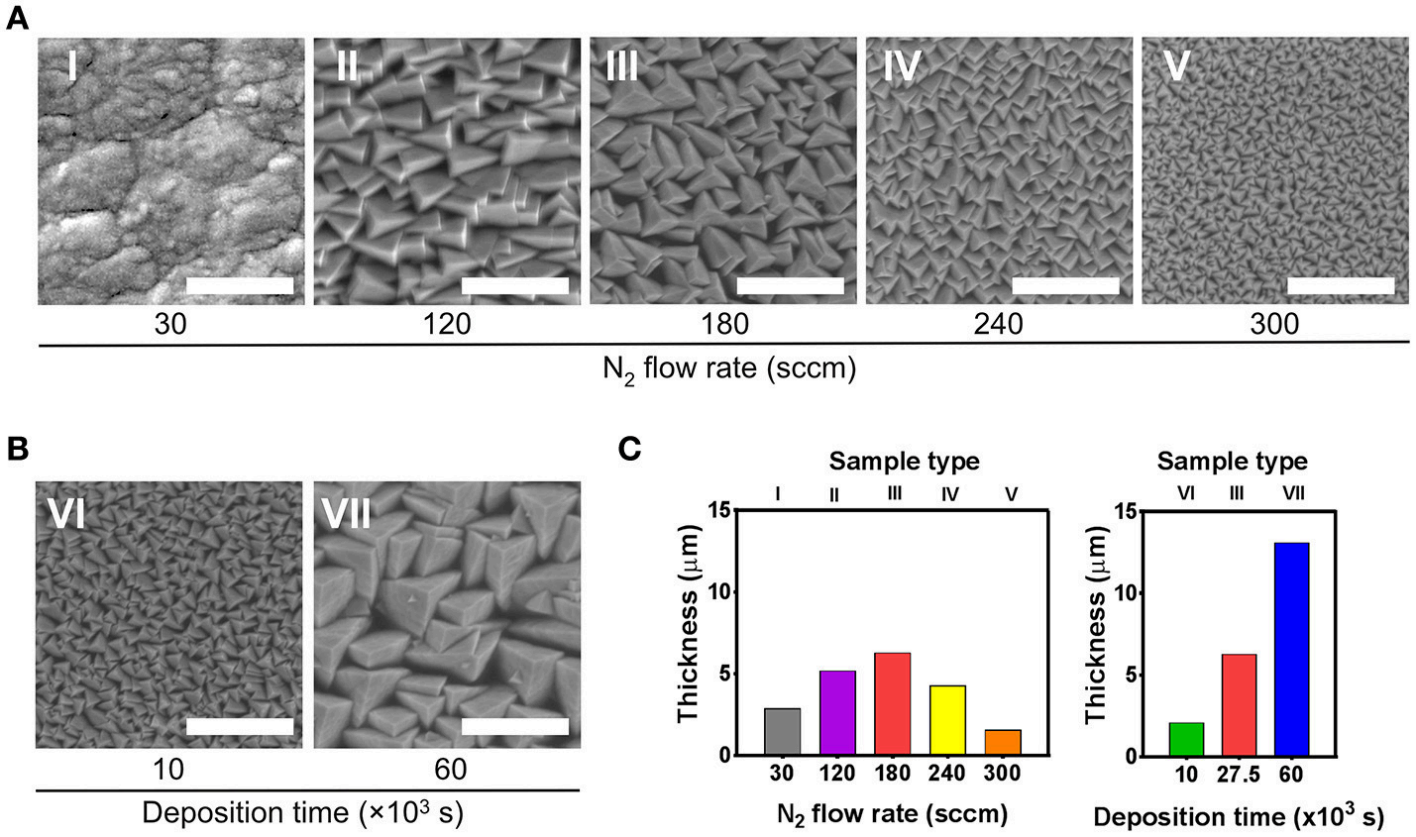
Scanning electron microscopy (SEM) analysis of the seven types of prepared TiN films (I–VII). **(A)** Effect of varying the N_2_ flow rate on film morphology. For this set of samples (I–V), the deposition time was kept at 27.5 × 10^3^ s. Films type I were used as smooth reference coatings. **(B)** Effect of varying the deposition time on film morphology, while keeping the N_2_ flow rate at 180 sccm. **(C)** Thickness of the films displayed in **(A,B)**. Scale bar in SEM images represents 1 μm.

The influence of deposition parameters on thickness and morphology of the TiN films was investigated by depositing films at different partial pressures of N_2_ while keeping the deposition time at 27.5 × 10^3^ s. The partial pressure of N_2_ in the deposition chamber was modified by varying the N_2_ flow rate. At the lowest flow rate, the TiN films displayed a relatively smooth surface (Figure 1A). These samples, designated as type I, were used as substrates for the planar reference coatings throughout the study. The remaining films, deposited at N_2_ flow rates ranging from 120 sccm and above, consisted of rough surfaces displaying pyramidal-like features whose lateral dimensions decreased at higher N_2_ flow rates (Samples II–V, Figure 1A). The porous TiN films comprise of a highly dense columnar-type structure, with pyramidal features at the top of the columns. The typical cross-section profile of porous TiN films is shown in the Supplementary Figure 1. Thickness displayed a non-monotonic dependence on the N_2_ flow rate (Figure 1C). The maximum film thickness was obtained at 180 sccm (sample type III), where the partial pressures of N_2_ and Ar are equal. Subsequently, the effect of deposition time on thickness and morphology of films was assessed by depositing films at a shorter and a longer time interval in relation to sample III (samples VI and VII). The column size (Figure 1B) as well as film thickness (Figure 1C) correlated directly to the deposition time.

### Assessment of the surface properties of BDD/TiN films

**Figure 2:**
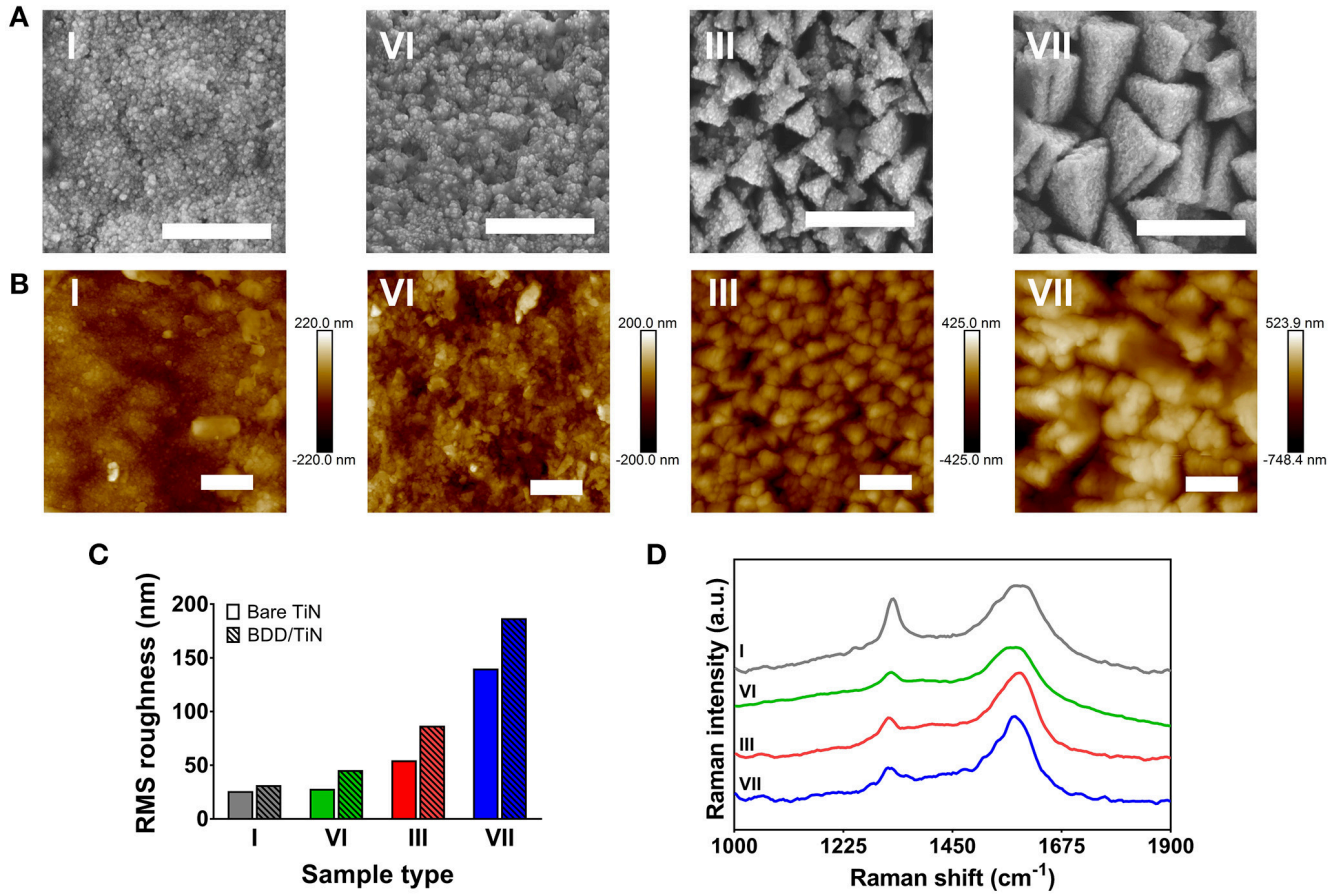
Surface analysis of four representative electrodes (type I, III, VI, and VII). **(A)** Scanning electron microscopy (SEM) images displaying the uniform coverage of the nanocrystalline diamond layer. Scale bar represents 1 μm. **(B)** Atomic force microscopy (AFM) height images. Scale bar represents 1 μm. **(C)** AFM surface roughness measurements from the films displayed in **(B)** before (bare TiN) and after diamond deposition (BDD/TiN). **(D)** 325-nm Raman spectra showing the diamond-related peak between 1,320 and 1,328 cm^−1^. The spectra have been offset for clarity.

Diamond thin-films were synthesized on all types of TiN coating (I–VII). SEM images (Figure 2A) along with AFM images (Figure 2B) show the morphology of BDD films grown on four representative substrates: smooth TiN (type I), and the three electrodes grown at a growth rate of 180 sccm (types III, VI, and VI). SEM images revealed that the BDD films had a uniform coverage on the TiN and displayed a nanocrystalline structure with a grain size of ~50 nm. Due to the electrode geometry, *in situ* BDD-film thickness measurements were not possible, however, deposition onto silicon substrates at identical conditions resulted in film thicknesses in the order of the grain size (i.e., ~50–70 nm).

The large-scale topography of the diamond films, as measured using an optical profilometer over an area of 220 × 280 μm^2^, was governed by grooves on the underlying TiAlV substrate, which are ~20 μm wide and up to 400 nm high. The roughness on this scale was around 200–350 nm and it was not significantly influenced by the TiN or the BDD coating. However, the small-scale topography measured by AFM over an area of 5 × 5 μm^2^ was mostly governed by the topography of the TiN pyramidal structure. The roughness of the diamond layer, which measured on a flat surface was around 30 nm, had only a minimal influence on the topography of the BDD/TiN films. Figure 2C shows the RMS surface roughness of the selected sample types before and after BDD deposition. As the electrode surface is not flat the error in the estimation of the roughness is ~20% when measured on different areas on the pin.

Raman spectroscopy confirmed the synthesis of diamond films in all TiN substrates. Figure 2D displays the Raman spectra of BDD films grown on the selected sample types. In all spectra, a shifted diamond peak is observed at 1,320–1,328 cm^−1^ as well as broad features related to sp^2^ at 1,360 and 1,585 cm^−1^, i.e., the D and G bands.

### Electrochemical characterization

**Figure 3:**
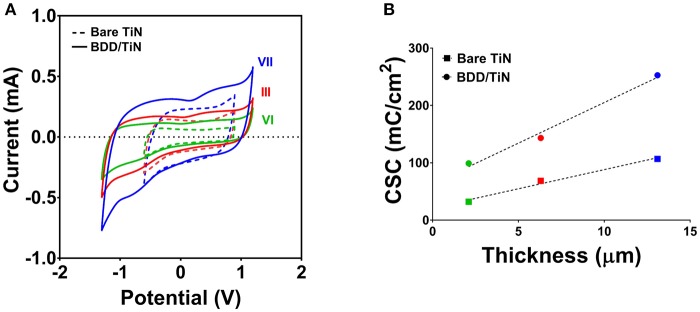
Cyclic voltammetry measurements of electrodes type III, VI, and VII before (bare TiN) and after BDD deposition (BDD/TiN). **(A)** Cyclic voltammograms showing that the safe potential limits are increased in BDD/TiN electrodes as compared to the bare counterparts. The potential limits are the same within the two electrode groups. **(B)** Cathodic charge storage capacity (CSC) of the electrodes as a function of the underlying TiN film thickness, showing that CSC-values were consistently increased after BDD deposition. Data from samples type III, VI, and VII are shown in red, green, and blue, respectively. Dotted lines represent the linear regression of the CSC-values.

The water window potentials were obtained by CV and their values were typically −0.6 to 0.9 V for TiN and −1.3 to 1.2 V for BDD/TiN electrodes (vs. Ag|AgCl). Table 1 summarizes the cathodic CSC-values which were obtained at a sweep rate of 0.05 V/s. The CSC obtained at a slow sweep rate gives an insight into the entire ESA of the porous electrodes. Due to the wide potential window brought by the BDD coating, the CSC of the BDD/TiN electrodes was consistently higher than the CSC of bare TiN electrodes. The CSC-values pre- and post-BDD deposition followed a linear relationship with a slope of 2.2, indicating that the CSC-values of the electrodes doubled upon BDD deposition (Supplementary Figure 2). It was also noticed that the CSC-values were drastically increased in all porous samples as compared to the smooth reference electrodes, for which the CSC-values were 0.36 and 7.74 mC/cm^2^, respectively. Table 1 includes the ESA/GSA ratio, which reflects the relative increase in the ESA for each of the porous samples, before and after BDD deposition. Figure 3A displays representative CV curves from electrodes type III, VI, and VI, before and after BDD deposition, where it is possible to observe the relative increase in the CSC when TiN films become deposited with BDD. As shown in Figure 3B, the CSC of these electrodes appears to increase linearly with an increase in the TiN coating thickness. The CSC-values fit a linear regression with a slope of 6.7 mC/cm^2^.μm for the bare TiN films (*r*^2^ = 0.98) and 14.2 mC/cm^2^.μm for the BDD/TiN films (*r*^2^ = 0.99), but the slopes are not significantly different (*P* > 0.05).

**Table 1:** Cathodic CSC of the bare TiN and BDD/TiN electrodes obtained at 0.05 V/s.

	**TiN**		**BDD/TiN**	
**Sample type**	**CSC (mC/cm^2^)**	**ESA/GSA ratio**	**CSC (mC/cm^2^)**	**ESA/GSA ratio**
I	0.36	–	7.74	–
II	54	150	105	14
III	69	190	143	19
IV	86	238	177	23
V	58	159	136	18
VI	32	89	99	13
VII	107	295	253	33

*The ESA/GSA ratios were calculated by dividing the CSC of each of the porous coatings by the CSC of the smooth reference coatings*.

**Figure 4:**
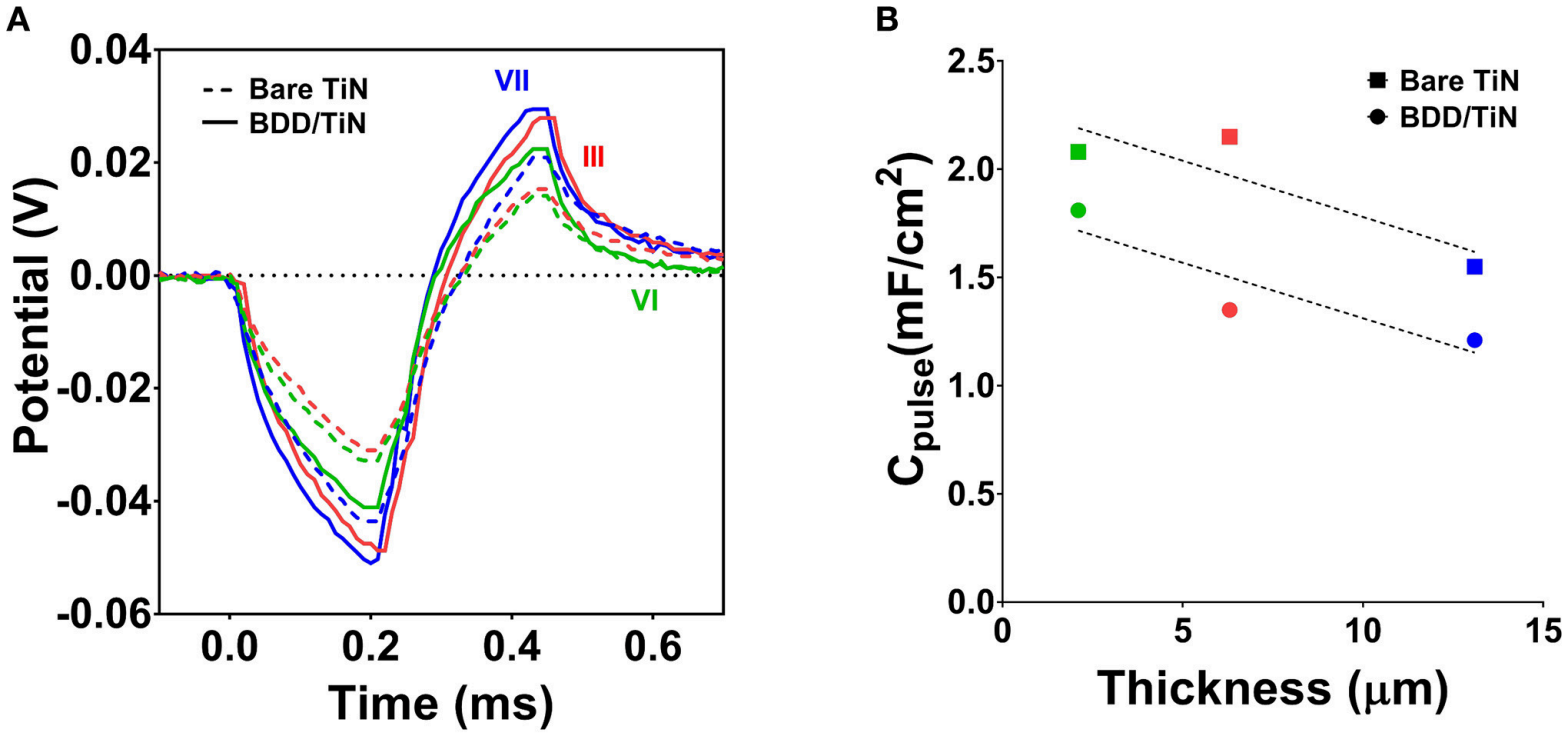
Voltage transient measurements of electrodes type III, VI, and VII before (bare TiN) and after BDD deposition (BDD/TiN). **(A)** The voltage transients of the BDD/TiN electrodes were larger than those of the bare TiN electrodes, evidencing a decrease in the pulsing capacitance (*C*_pulse_). **(B)** *C*_pulse_ of the electrodes as a function of the underlying TiN film thickness, showing that *C*_pulse_-values were decreased after BDD deposition. Data from samples type III, VI, and VII are shown in red, green, and blue, respectively. Dotted lines represent the linear regression of the *C*_pulse_-values.

Table 2 summarizes the *C*_pulse_ for all electrode types, which was derived from the VT measurements. Except for electrode type I, the *C*_pulse_ of the BDD/TiN electrodes was consistently lower than the *C*_pulse_ of the corresponding TiN electrodes. Figure 4A shows representative VT measurements on electrodes type III, VI, and VII, before and after BDD coating. As compared to the bare TiN electrodes, the lower *C*_pulse_ of BDD/TiN electrodes leads to larger electrode potentials. However, as the safe potential window for BDD is larger than for TiN, the amount of charge that can safely be injected will be higher for BDD/TiN than for TiN. *C*_pulse_ displays a negative trend for both TiN and BDD/TiN electrodes as a function of TiN coating thickness (Figure 4B). The *C*_pulse_-values fit similar linear regressions, with a slope of −0.051 mF/cm^2^.μm for the bare TiN films (*r*^2^ = 0.82) and −0.052 mF/cm^2^.μm for the BDD/TiN films (*r*^2^ = 0.77).

**Table 2:** Pulsing capacitance of the bare TiN and BDD/TiN electrodes.

**Sample type**	**TiN (mF/cm^2^)**	**BDD/TiN (mF/cm^2^)**
I	0.024	0.059
II	1.6	1.6
III	2.2	1.4
IV	1.9	1.5
V	2.0	1.5
VI	2.1	1.8
VII	1.6	1.2

*Values are derived from the voltage transient measurements*.

**Figure 5:**
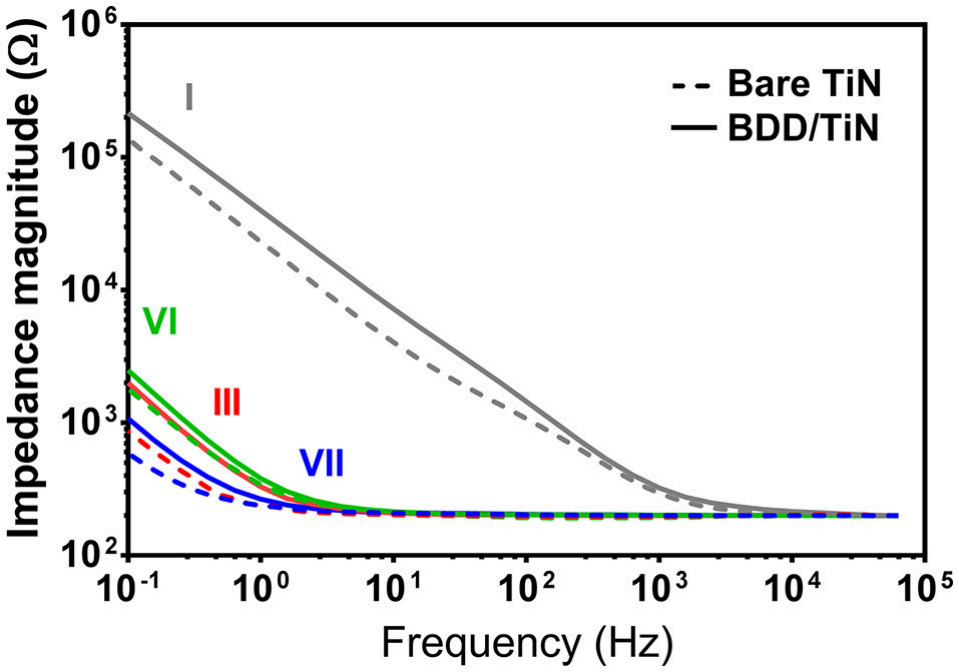
Impedance magnitude spectra of electrodes type I, III, VI, and VII before (bare TiN) and after BDD deposition (BDD/TiN). Data from electrodes type I, III, VI, and VII are shown in gray, red, green, and blue, respectively.

As anticipated, increased film porosity significantly reduced the impedance of the electrodes (Figure 5). The impedance magnitudes of the TiN and BDD/TiN porous electrodes were only different at frequencies below 10 Hz. The greatest difference in impedance magnitude between TiN and BDD/TiN was at 100 mHz, where the BDD electrodes consistently had a higher impedance than the TiN electrodes. The impedance magnitude of both BDD and TiN electrodes decreased with increasing thickness. The lowest impedance magnitude for TiN with and without BDD coating were obtained using the electrode with the thickest TiN coating (type VII).

## Discussion

A range of porous TiN films, to be used as templates, was deposited onto test electrodes by means of a physical vapor deposition. The smooth TiN reference films were fabricated using a low partial pressure of N_2_ to ensure a stoichiometric Ti/N ratio below 0.6, which has been shown to be unfavorable for columnar growth (Igasaki et al., 1978; Cunha et al., 2009). Formation of the columnar, highly porous structures was favored using N_2_ flow rates of 120 sccm and above. For the set of samples II–V, higher N_2_ flow rates resulted in decreased film columnar width. This effect has been associated to a reduced mobility of the deposited atoms as a consequence of a weakening in the argon bombardment (Arshi et al., 2012). Furthermore, increasing the N_2_ flow poisons the Ti targets to the extent where the entire target surface is covered in TiN. This effect decreases the deposition rate as the sputter yield is lower for TiN than for Ti (Berg and Nyberg, 2005), which also results in thinner films with smaller columns. The deposition rate appeared to be maximal at a N_2_ flow rate of 180 sccm, where the N_2_ flow rate equals that of Ar and it is assumed that deposition occurs at a stoichiometric Ti/N ratio. TiN films with stoichiometric Ti/N composition are usually preferred due to their optimal mechanical and electrical properties (Kang and Kim, 1999; Martinez et al., 2014). Samples III, VI, and VII were therefore deposited keeping the N_2_ flow constant and varying the deposition time to obtain porous films with similar crystalline composition but different thickness. A longer deposition time increased the columnar width, which is a result of the competitive growth where some columns grow at the expense of others. Such a growth is typically observed for coatings deposited at a relatively low temperature compared to the melting temperature of the coating material (Ohring, 2002). For the TiN films, increasing the N_2_ flow (in samples I–III) led to higher thickness and higher porosity, which was reflected as an increased ESA/GSA ratio. Further increase in N_2_ (sample IV) still gave an increase in area due to increased porosity, although the thickness was smaller. Going to higher N_2_ the growth rate was slower, so that the lower thickness dominated over the increased porosity and an overall decrease in the ESA/GSA was obtained. For the samples grown at constant gas flow rate (III, VI, and VII), film thickness, and ESA/GSA ratio were directly correlated.

Surface analysis of the BDD/TiN samples by SEM and AFM revealed homogeneous and high quality BDD films. The overall structure and topography of the films appeared similar to the bare TiN samples, suggesting that CVD deposition did not significantly affect the morphology of the TiN template. The uniform coverage of diamond crystallites indicates a highly cohesive diamond film. This is in agreement with previous studies, which have shown that TiN possesses several favorable properties for nucleation and growth of good quality CVD diamond films, including low diffusivity of carbon, compatible interatomic potential, and small lattice mismatch (Weiser et al., 1992; Kumar et al., 1997; Polini et al., 2006). In addition, since TiN exhibits a moderate interface reactivity, its surface is stable under high-temperature diamond–CVD deposition (Contreras et al., 2000). Moreover, given the similar thermal expansion coefficient of both materials, the interlayer stresses are minimal, which ensures the synthesis of highly adherent diamond layers (Kumar et al., 1997). Although we did not encounter any evidence of cracks or film delamination, future studies should further investigate the nature the BDD/TiN interlayer by appropriate techniques, as for instance transmission electronic microscopy (TEM). Concerning the Raman analysis, the shifts in diamond's Raman peak can be related to a variation of stress in layers, nonetheless its shift to lower wavenumbers is associated with increasing B incorporation in the lattice (Prawer and Nemanich, 2004). It is worth noting that the spectra in Figure 2D are representative only, i.e., the ratio of sp^3^/sp^2^ changes with measurement position. This apparent variation in sp^3^/sp^2^ is related to the fact that the BDD coating is very thin and therefore the grain boundary content is high.

The superior ESA of the TiN coatings used as porous templates is evident from the high ESA/GSA ratios and the drastic reduction in the impedance magnitudes. Thicker films had a consistently higher CSC, suggesting that pores extend into the entire depth of the coating, which is in agreement with previous studies (Norlin et al., 2005; Cunha et al., 2009). BDD deposition onto the TiN coatings significantly increased the CSC of the electrodes due to the wide potential window of diamond. The linear correlation between the CSC of the TiN and the BDD/TiN electrodes indicates that the diamond films did not block the pores. On the other hand, the *C*_pulse_ showed a negative correlation with film thickness, as the highest *C*_pulse_-values were obtained with thinner coatings and smaller column width. The BDD films might therefore cause narrowing of the pores, with a consequent increase in the pore resistance. This effect decreases the pore depth that can be used under pulsing conditions (Cogan, 2008). Thus, increasing the coating thickness beyond a certain level would be less advantageous for electrical stimulation purposes. While *C*_pulse_ is decreased for BDD/TiN as compared to bare TiN electrodes, it is important to view this result in the light of the wide safe potential window of BDD (Garrett et al., 2011). The decrease in *C*_pulse_ after depositing BDD ranged from 67% to <1%, while the cathodic potential limit was more than doubled (−0.6 V for TiN vs. −1.3 for BDD/TiN). This means that the amount of charge that can be injected without reaching unsafe potentials is doubled by applying a BDD thin-film onto a porous TiN coating. It is important to view these results in the light of the intended application of the BDD/TiN electrodes, which is *in vivo* chronic neurostimulation. It has been shown that the stimulation performance of TiN electrodes deteriorates after implantation (Meijs et al., 2015a, 2016b,c). This is not the case for BDD electrodes, which display a remarkable resistance to protein biofouling (Trouillon and O'Hare, 2010; Alcaide et al., 2016a; Meijs et al., 2016a). Nevertheless, protein adsorption is influenced by surface topography, which warrants further investigation of the electrochemical performance of porous BDD/TiN electrodes in protein-rich environments.

The relatively low *C*_pulse_ and high impedance shown by the smooth BDD electrodes was evident and corresponds well to what has been shown in previous studies (Garrett et al., 2011; Meijs et al., 2013). Remarkably, the electrochemical performance of BDD displayed a significant improvement thanks to the large ESA gained by using the porous TiN templates. As previous studies have shown, other porous templates have been instrumental in enhancing the electrochemical performance of BDD (Bonnauron et al., 2008; Kiran et al., 2013; Hébert et al., 2014a). A notable example is the growth of BDD on vertically aligned nanotubes, which has shown to increase the CSC of BDD up to 10 mC/cm^2^ (Piret et al., 2015). The BDD/TiN electrodes in the current, however, displayed CSC-values up to 253 mC/cm^2^ for the type VII electrode. Furthermore, while the use of carbon nanotube based materials for human implants remains controversial due to evidence of cytotoxic effects (Smart et al., 2006; Liu et al., 2013), both TiN and BDD have demonstrated low risk of cytotoxicity and excellent biocompatibility in diverse applications. TiN is well known for improving the electrochemical and biocompatibility properties of various materials (Subramanian et al., 2011) and represents one of the coatings with a long history of clinical use for orthopedic implants (Gotman et al., 2014; van Hove et al., 2015). Data from implantation studies revealed that BDD electrodes are associated with no signs of chronic inflammation and a very thin fibrous capsule (Alcaide et al., 2016b). Taken together, these results indicate that this novel type of combined coating may be used to fabricate safe implants for clinical use. Furthermore, the method can be easily scaled-up, making the production process fast and cost-effective (Taylor et al., 2014, 2018). The production process is reproducible and clean, as both coatings are deposited under vacuum conditions. Overall, these factors make this novel type of coating particularly attractive for the development of commercially viable electrodes for neural interfacing.

To achieve an increased charge injection (*Q*_inj_), the production parameters are of critical importance, as the extra coating increases the pore resistance, which may deteriorate Q_inj_. This study suggests that specific deposition parameters are optimal for stimulation electrodes, as increased thickness and N_2_ flow only result to a certain extent in larger *C*_pulse_ and *Q*_inj_. The data suggests that BDD deposited onto thinner coatings with smaller columnar size results in better stimulation performance. A thicker coating, however, results in a high CSC and low impedance, which could be exploited for other purposes, such as electrical and electrochemical sensing. This highlights the versatility of the novel coating combination presented in this work.

## Conclusion

In this work, we have fabricated a range of BDD/porous TiN electrodes with very high surface area, which exhibit a broad safe potential window and CSC-values which are superior to those reported in the literature for porous BDD electrodes. Electrodes with more porous and thick coatings were associated with higher CSC and lower impedance magnitudes, but the relatively limited *C*_pulse_ would make them more suited for sensing applications. On the other hand, relatively higher *C*_pulse_ were obtained with thinner films with small column size, which would result more favorable for stimulation applications. Although BDD/TiN electrodes displayed a higher impedance magnitude and a lower *C*_pulse_ as compared to the bare TiN electrodes, the wider potential window likely allows for higher *Q*_inj_ without reaching unsafe potentials. These remarkable improvements, together with the known mechanical stability, resistance to biofouling and long-term *in vivo* stability of BDD films, makes this coating combination an ideal candidate for development of reliable devices for chronic neural interfacing. This novel type of coating is particularly attractive for the development of commercially viable electrodes due to the simplicity and the scalability of the approach.

## Author contributions

AT, MN, and CP conceived and designed the study. SM, MM, SS, and KR prepared the samples. MM, SS, KR, LF, LK, and AT obtained the data and performed the surface analysis of the films. SM and VP acquired and analyzed the electrochemistry data under supervision of NR. SM and CP drafted the manuscript and designed the figures. All authors contributed to the critical revision of the draft and approved the submitted version.

### Conflict of interest statement

The authors declare that the research was conducted in the absence of any commercial or financial relationships that could be construed as a potential conflict of interest.
