# Supplementary material for: Diamond/Porous Titanium Nitride Electrodes With Superior Electrochemical Performance for Neural Interfacing
Source: Front Bioeng Biotechnol. 2018 Nov 15;6:171. doi: 10.3389/fbioe.2018.00171 (PMC6262293; doi:10.3389/fbioe.2018.00171)
Supplement: Supplementary file 1 [file Presentation_1.pdf]

Supplementary Material

Diamond/Porous Titanium Nitride Electrodes with Superior Electrochemical Performance for Neural Interfacing

Suzan Meijs, Matthew McDonald, Søren Sørensen, Kristian Rechendorff, Ladislav Fekete, Ladislav Klimša, Václav Petrák, Nico Rijkhoff, Andrew Taylor, Miloš Nesládek, and Cristian P Pennisi *

*** Correspondence:** cpennisi@hst.aau.dk

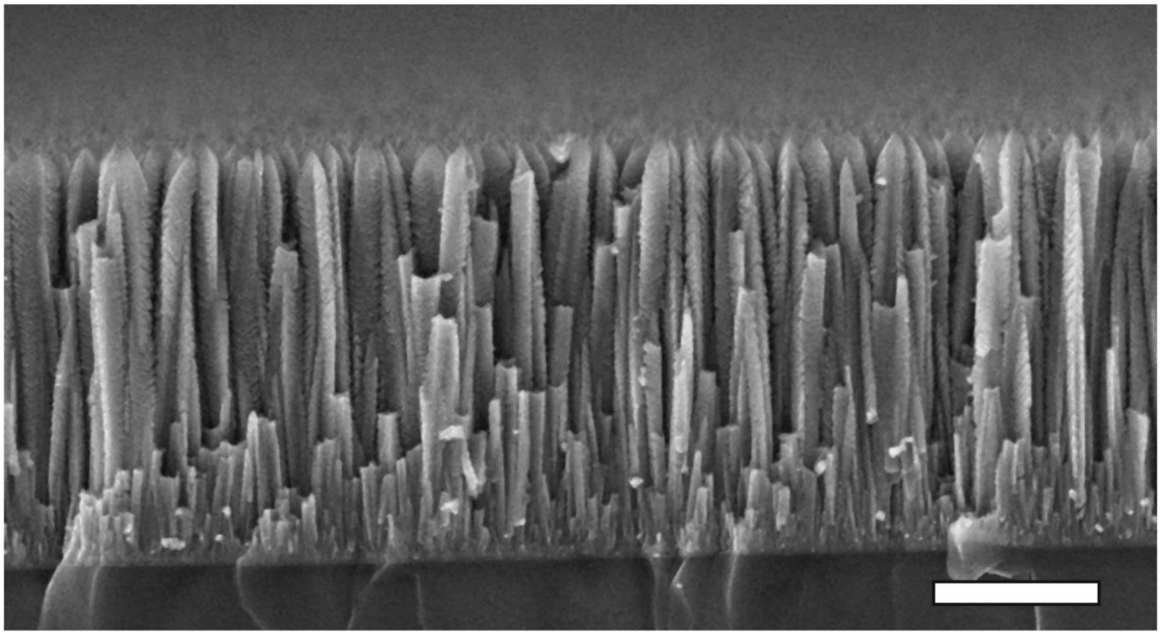

Supplementary Figure 1. Cross sectional SEM image of a bare TiN coating showing the high surface area and typical columnar profile of the films. The displayed image corresponds to an electrode type VI. Scale bare denotes 1 µm.

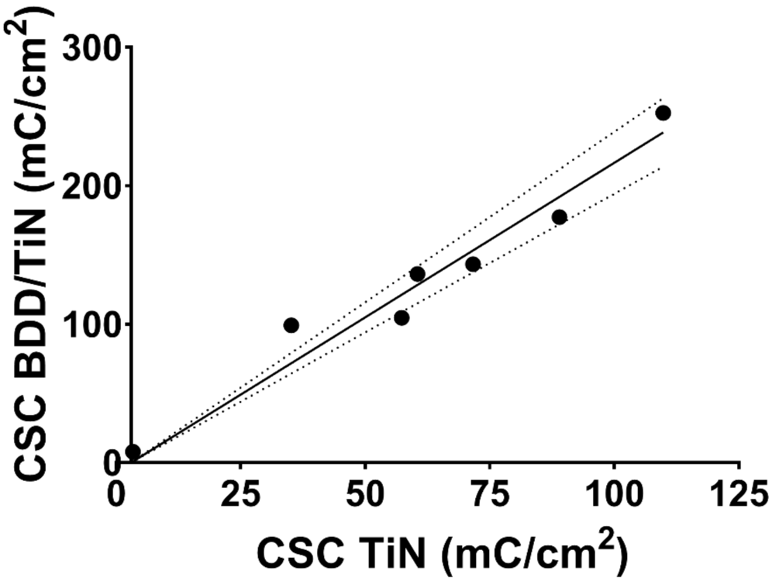

Supplementary Figure 2. Correlation between the CSCs of TiN and BDD/TiN electrodes. There is a linear relationship represented by $y = 2.2\ x$ (r^2=0.95). The stapled line represents the 95% confidence interval.
